# MR enterography radiologic ulcers in newly diagnosed ileal Crohn disease in children: frequency, inter-radiologist agreement, and clinical correlation

**DOI:** 10.1007/s00247-024-06056-7

**Published:** 2024-09-18

**Authors:** Andrew Palmer, Alexander J. Towbin, Christopher G. Anton, Murat Kocaoglu, Bin Zhang, Kaitlin Whaley, Pradipta Debnath, Jonathan R. Dillman

**Affiliations:** 1 Department of Radiology, Cincinnati Children’s Hospital Medical Center, 3333 Burnet Avenue, Cincinnati, OH 45229 USA; 2 Department of Radiology, University of Cincinnati College of Medicine, Cincinnati, OH USA; 3 Division of Biostatistics and Epidemiology, Cincinnati Children’s Hospital Medical Center, Cincinnati, OH USA; 4 Division of Gastroenterology, Hepatology, and Nutrition, Cincinnati Children’s Hospital Medical Center, Cincinnati, OH USA

**Keywords:** Children, Crohn disease, Inter-radiologist agreement, MR enterography, Ulcers

## Abstract

**Background:**

Radiologic ulcers are increasingly recognized as an imaging finding of bowel wall active inflammation in Crohn disease (CD).

**Objective:**

To determine the frequency of ulcers at MR enterography (MRE) in children with newly diagnosed ileal CD, assess agreement between radiologists, and evaluate if their presence correlates with other imaging and clinical features of intestinal active inflammation.

**Materials and methods:**

This retrospective study included 108 consecutive pediatric patients (ages 6–18 years) with newly diagnosed ileal CD that underwent clinical MRE prior to treatment initiation between January 2021 and December 2022. MRE examinations were independently reviewed by three pediatric radiologists who indicated the presence vs. absence of ulcers, ulcer severity (categorical depth), and ulcer extent (categorical number of ulcers). Maximum bowel wall thickness and length of disease were measured and averaged across readers. Patient demographics and clinical inflammatory markers were documented from electronic health records. Inter-radiologist agreement was assessed using Fleiss’ kappa (*k*) statistics. Student’s *t*-test was used to compare continuous variables.

**Results:**

Mean patient age was 13.9 years (67 [62%] boys). Radiologic ulcers were recorded in 64/108 (59.3%) cases by reader 1, 70/108 (64.8%) cases by reader 2, and 49/108 (45.4%) cases by reader 3 (*k* = 0.36). Based on majority consensus, radiologic ulcers were present in 60/108 (55.6%) participants. Inter-radiologist agreement for ulcer severity was *k* = 0.23, while ulcer extent was *k* = 0.66. There were significant differences in C-reactive protein, erythrocyte sedimentation rate, fecal calprotectin, albumin, maximum bowel wall thickness, and length of disease between patients without and with radiologic ulcers (*P* < 0.05). The sensitivity and specificity of MRE for detecting endoscopic ulcers were 66.7% (95% CI, 52.1–79.2%) and 69.2% (95% CI, 48.2–85.7%), respectively.

**Conclusion:**

Radiologic ulcers are visible in children with newly diagnosed ileal CD, although inter-radiologist agreement is only fair. The presence of ulcers is associated with clinical laboratory inflammatory markers as well as other MRE findings of disease activity and is an additional imaging finding that can be used to evaluate intestinal inflammation.

**Supplementary Information:**

The online version contains supplementary material available at 10.1007/s00247-024-06056-7.

## Introduction

MRI of the bowel, or MR enterography (MRE), is commonly used to evaluate children and adults with known or suspected Crohn disease (CD) [1, 2]. This imaging test can noninvasively detect and characterize intestinal active inflammation, including disease location and severity, as well as identify a variety of disease-related complications, including strictures, fistulas, and abscesses [1, 2].

Conventional MRE features of bowel wall active inflammation include mural thickening, postcontrast hyperenhancement, and intramural inflammation, including increased signal intensity on both T2-weighted and high *b*-value diffusion-weighted imaging [1–3]. Increasingly, mucosal ulcers also have been described as a radiologic finding of moderate to severe intestinal active inflammation [4–6]. The presence of radiologic ulcers has been shown to be associated with the presence of endoscopic ulcers [5, 6]. However, there is a paucity of data demonstrating the frequency of ulcers by imaging and if they are associated with other clinical or MRI findings of intestinal active inflammation. Furthermore, it remains unknown if this imaging feature can be reliably detected with satisfactory inter-radiologist agreement, particularly in children.

The purpose of this study was to determine the frequency of ulcers at MRE in children with newly diagnosed ileal Crohn disease, assess agreement between radiologists for this imaging feature, and evaluate if their presence is associated with other imaging and clinical features of intestinal active inflammation.

## Methods

This retrospective study was institutional review board-approved and performed in a Health Insurance Portability and Accountability Act-compliant manner. The need for informed consent was waived.

**Fig. 1:**
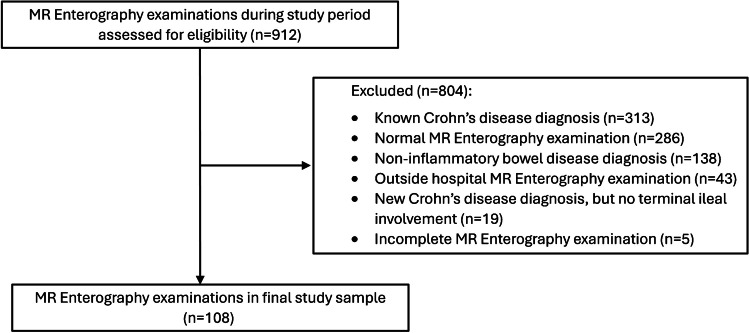
Participant flow diagram

Using a review of institutional electronic health records, consecutive pediatric patients (ages 6 years to 18 years) with newly diagnosed ileal CD that underwent a clinical MRE examination prior to medical treatment initiation between January 1, 2021, and December 31, 2022, were identified. Patients without a clinical diagnosis of CD in the electronic health record, including endoscopic and histologic confirmation of ileal CD, were excluded as were patients with incomplete MRE examinations and MRE examinations performed at outside institutions. A participant flow diagram is presented in Fig. 1.

For each participant included in the final study sample, demographic and anthropometric data were recorded. Clinical markers of intestinal active inflammation also were recorded from the time of ileal CD diagnosis, including C-reactive protein (CRP), erythrocyte sedimentation rate (ESR), albumin, hematocrit, and fecal calprotectin. All laboratory data was obtained within 3 months of the MRE examination. In patients that underwent repeated laboratory testing, the values closest to the date of the MRE examination were documented. Clinical MRE and endoscopy reports were used to phenotype participants based on disease location and the presence of stricturing and internal penetrating disease. In addition, clinical endoscopy reports were used to document the diagnostic adequacy and the presence of ulcers (e.g., superficial, deep, linear) involving the terminal ileum; participants with erosions and aphthous lesions by endoscopy were considered to be negative for ulcers for the purposes of this study.

Pertinent clinical MRE examinations were independently reviewed by three fellowship-trained, board-certified pediatric radiologists (26 years, 16 years, and 4 years post-fellowship experience). Study radiologists were blinded to one another and were not provided any additional clinical (e.g., laboratory or endoscopic) data. All MRE examinations included a combination of axial and coronal single-shot fast spin-echo without and with fat suppression, axial diffusion-weighted, and axial and coronal contrast-enhanced T1-weighted sequences based on our institutional clinical MRE imaging protocol (which remained unchanged during the study period) (Supplemental Table). MRE images were acquired following the ingestion of oral contrast material (Breeza; Beekley Corporation, Bristol, CT) using weighted-based dosing (20 ml/kg, up to 1,000 ml).

**Fig. 2:**
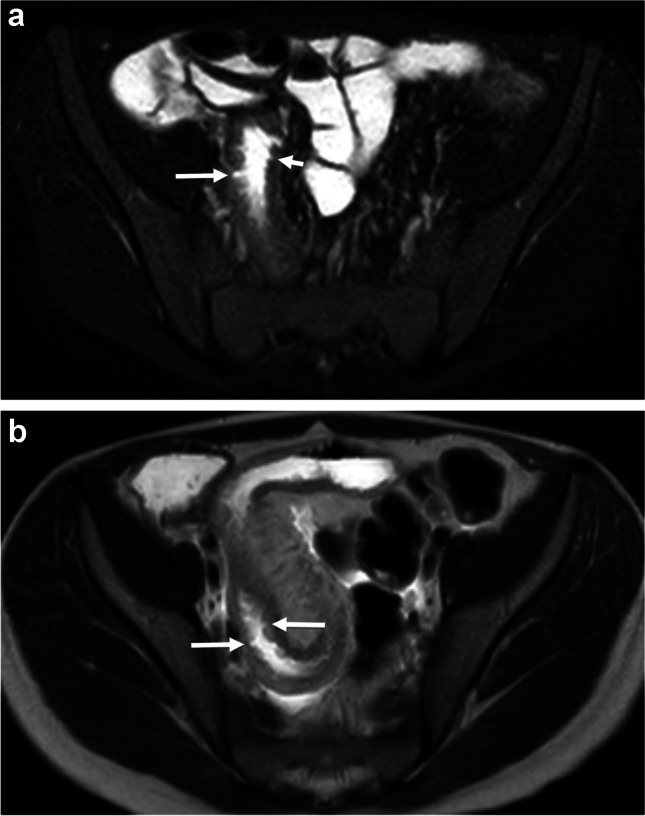
Examples of radiologic ulcers from two different children with newly diagnosed ileal Crohn disease. **A** Axial T2-weighted single-shot fast spin-echo MR image with fat suppression from a 12-year-old boy shows both deep (*long arrow*) and superficial (*short arrow*) radiologic ulcers. The terminal ileum appears thick-walled with mural edema. **B** Axial T2-weighted single-shot fast spin-echo image without fat suppression from a different 12-year-old boy shows multiple superficial radiologic ulcers (*arrows*). The terminal ileum appears thick-walled with mural edema. All three study radiologists indicated the presence of radiologic ulcers in these two children as well as agreed on severity (i.e., ulcer depth)

**Fig. 3:**
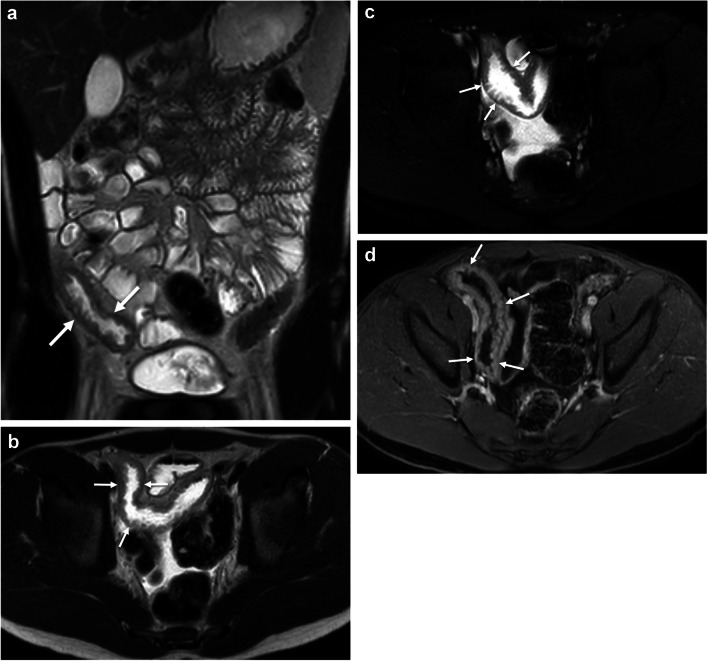
A 15-year-old with a family history of Crohn disease and new perianal drainage. **A** Coronal T2-weighted single-shot fast spin-echo, (**B**) axial T2-weighted single-shot fast spin-echo, (**C**) axial T2-weighted single-shot fast spin-echo fat-saturated, and (**D**) axial postcontrast T1-weighted MR images show findings consistent with ileal Crohn disease, including multiple radiologic ulcers (*arrows*) of variable depth and severe in extent (6 or more visible ulcers)

Each study radiologist assessed the terminal ileum (defined as the distal 15 cm of the small bowel, including the ileocecal valve, for the purposes of this investigation) and documented the following MRI findings for each MRE examination:

- Presence vs. absence of radiologic ulcers (yes/no), defined as a mucosa-based defect in the bowel wall filled with oral contrast material or enteric contents (Figs. 2 and 3);
- Severity of radiologic ulcers – classified based on the most severe ulcer (none, depth < 50% bowel wall thickness, or depth > 50% bowel wall thickness, scored as 0–2);
- Extent of radiologic ulcers (none, mild [1–2 ulcers], moderate [3–5 ulcers], or severe [6 or more ulcers], scored as 0–3);
- Maximum single bowel wall thickness in mm; and
- Length of disease in cm (maximum 15 cm).

Multiple unique examples of radiologic ulcers meeting the above study definitions on both noncontrast and contrast-enhanced MR sequences were assembled by a fourth radiologist (15 years post-fellowship experience) and shared with the study radiologists prior to their imaging review to serve as a training aid.

### Statistical analyses

Continuous data were summarized as means and standard deviations, while categorical data were summarized as counts and percentages. Student’s *t*-test (two-sided) was used to compare continuous variables between groups, while Fisher’s exact test was used to compare categorical variables between groups. Pearson’s and Spearman’s rank-order correlation coefficients were used to evaluate associations between variables, as appropriate. The consensus among the three study radiologists was used to categorize patients into two groups, patients without and patients with radiologic ulcers (with either all three or two of three study radiologists agreeing on their absence vs. presence). Mean maximum bowel wall thickness, mean length of disease, and mean extent of ulcers among the three study radiologists were used for correlation testing. The diagnostic performance (sensitivity, specificity, positive predictive value, and negative predictive value) of MRE for diagnosing ulcers (based on study radiologist consensus) was also determined using the presence of ulcers at clinical endoscopy as the reference standard.

Inter-radiologist agreement was assessed using Fleiss’ kappa (*k*) statistics, with weighted kappa statistics calculated for ordinal variables (e.g., extent of ulcers). Kappa statistics were interpreted as follows: < 0 = no agreement; 0–0.20 = slight agreement, 0.21–0.40 = fair agreement, 0.41–0.60 = moderate agreement, 0.61–0.80 = substantial agreement, and 0.81–1 = almost perfect agreement [7].

A *P*-value < 0.05 was considered significant for all inference testing. Statistical analyses were performed using MedCalc® Statistical Software version 20.111 (MedCalc Software Ltd, Ostend, Belgium).

## Results

### Study sample

One hundred and eight pediatric patients with newly diagnosed ileal CD were included in our final study sample. Mean (standard deviation) participant age was 13.9 (3.3) years (interquartile range, 11.0–17.0 years); 67 (62%) were boys. Participant characteristics, including demographic, anthropometric, and clinical laboratory data, are presented in Table 1.

**Table 1:** Participant characteristics, including in Crohn disease patients without and with radiologic ulcers based on consensus interpretation of three study radiologists. Data are presented as means and standard deviations or counts and percentages. Significant differences between patients without and with radiologic ulcers are bolded

Variable	Overall study sample (*n* = 108)	Patients without radiologic ulcers (*n* = 48)	Patients with radiologic ulcers (*n* = 60)	*P*-value*
Age (years) [interquartile range]	13.9 ± 3.3 [11.0–17.0]	14.3 ± 3.3 [13.3–15.2]	13.5 ± 3.2 [12.7–14.4]	0.26
Sex	F = 41 (38%) M = 67 (62%)	F = 17 (35%) M = 31 (65%)	F = 24 (40%) M = 36 (60%)	0.69
BMI (kg/m^2^)	20.1 ± 5.9	21.0 ± 6.8	19.4 ± 5.0	0.18
CRP (mg/ml)	3.7 ± 3.7	2.7 ± 3.2	4.4 ± 3.8	0.01
ESR (mm/h)	29.8 ± 22.1	23.4 ± 20.5	34.8 ± 22.2	0.008
Fecal calprotectin (µg/g)	2,458 ± 2,102	1,818 ± 1,908	2,922 ± 2,132	0.01
Hematocrit (%)	37.6 ± 4.7	38.1 ± 4.1	37.1 ± 5.1	0.25
Albumin (mg/dl)	3.4 ± 0.6	3.6 ± 0.6	3.3 ± 0.6	0.02
Maximum bowel wall thickness (mm)	5.6 ± 1.9	4.5 ± 1.7	6.4 ± 1.6	< 0.0001
Length of disease (cm)	9.2 ± 5.6	5.2 ± 3.6	12.5 ± 4.9	< 0.0001
Location of disease (Montreal classification)^a^	Ileum (L1) = 45 (41.7%) Colon (L2) = 2 (1.9%) Ileocolonic (L3) = 61 (56.4%) Upper tract (L4) = 59 (55.1%)	Ileum (L1) = 19 (39.6%) Colon (L2) = 2 (4.2%) Ileocolonic (L3) = 27 (56.2%) Upper tract (L4) = 25 (52.1%)	Ileum (L1) = 26 (43.3%) Colon (L2) = 0 (0%) Ileocolonic (L3) = 34 (56.7%) Upper tract (L4) = 34 (56.7%)	0.55
Stricture	No = 101 (93.5%) Yes = 7 (6.5%)	No = 47 (98%) Yes = 1 (2%)	No = 55 (92%) Yes = 5 (8%)	0.22
Internal penetrating disease	No = 91 (84.3%) Yes = 17 (15.7%)	No = 44 (92%) Yes = 4 (8%)	No = 50 (83%) Yes = 10 (17%)	0.26

*BMI*, body mass index; *CRP*, C-reactive protein; *ESR*, erythrocyte sedimentation rate; *F*, female; *M*, male

^*^P-value comparing radiologic ulcers vs. no radiologic groups using Student’s *t*-test or Fisher’s exact test, as appropriate

^a^L4 modifier can be in addition to L1-L3 classification when concomitant upper gastrointestinal tract involvement is present

### Frequency of radiologic ulcers and ulcer characteristics

Radiologic ulcers were recorded in 64 of 108 (59.3%) examinations by reader 1, 70 of 108 (64.8%) examinations by reader 2, and 49 of 108 (45.4%) examinations by reader 3. Inter-radiologist agreement was fair with *k* = 0.36 [95% CI, 0.25–0.47]. Based on majority consensus, radiologic ulcers were present in 60/108 (55.6%) participants.

Ulcer severity was scored as superficial vs. deep in 40 vs. 24 examinations by reader 1, 27 vs. 43 examinations by reader 2, and 21 vs. 28 examinations by reader 3 (*k* = 0.23 [95% CI, 0.12–0.34]).

Ulcer extent was scored as none, mild, moderate, or severe in 44, 11, 23, and 30 examinations by reader 1; 38, 28, 31, and 11 examinations by reader 2; and 59, 9, 17, and 23 examinations by reader 3 (*k* = 0.66 [95% CI, 0.56–0.74]).

### Associations between radiologic ulcers and clinical markers of active inflammation

There were significant differences in CRP (*P* = 0.01), erythrocyte sedimentation rate (*P* = 0.008), fecal calprotectin (*P* = 0.01), and albumin (*P* = 0.02) between patients without and with radiologic ulcers. These results are presented in Table 1.

There was a significant negative correlation between extent of ulcers (i.e., number of visible ulcers) and albumin (*r* = -0.25 [95% CI, -0.43-(-)0.06]; *P* = 0.01). No other laboratory values correlated with ulcer extent or severity (*P*-values > 0.05) (Table 2).

**Table 2:** Associations between MRI findings, including severity and extent of radiologic ulcers, and clinical laboratory data. Pearson’s or Spearman’s rank-order correlation (*) coefficients (*r*) are presented, with 95% confidence intervals (in parentheses) and *P*-values (in brackets)

	ESR	CRP	Fecal calprotectin	Albumin	Hematocrit
Maximum bowel wall thickness (mm)	0.13 (-0.06–0.32) [0.17]	0.11 (-0.09–0.29) [0.28]	0.11 (-0.10–0.31) [0.32]	-0.01 (0.20–0.19) [0.95]	-0.05 (-0.24–0.15) [0.64]
Length of disease (cm)	0.02 (-0.20–0.20) [0.84]	0.12 (-0.07–0.30) [0.22]	0.04 (-0.18–0.24) [0.74]	0.06 (-0.13–0.25) [0.54]	0.06 (-0.14–0.25) [0.57]
Extent of ulcers (0–3)*	0.09 (-0.1–0.28) [0.37]	0.01 (-0.18–0.21) [0.89]	0.11 (-0.11–0.32) [0.30]	-0.25 (-0.43–0.06) [0.01]	-0.11 (-0.24–0.15) [0.26]
Severity of ulcers (0–2)*	0.06 (-0.14–0.25) [0.56]	-0.03 (-0.23–0.17) [0.76]	0.04 (-0.18–0.25) [0.73]	-0.18 (0.36–0.02) [0.07]	-0.17 (-0.35–0.03) [0.09]

*CRP*, C-reactive protein; *ESR*, erythrocyte sedimentation rate

### Associations between radiologic ulcers and other MRE findings of active inflammation

There were significant differences in maximum single wall bowel thickness (*P* < 0.0001) and length of disease (*P* < 0.0001) between patients without and with radiologic ulcers. These results are presented in Table 1.

There were no significant correlations between laboratory values and either maximum single wall bowel thickness or length of disease (*P*-values > 0.05) (Table 2).

### Diagnostic performance of MRE for detecting endoscopic ulcers

The terminal ileum was able to be adequately evaluated by endoscopy in 78/108 (72%) of participants, while it was incompletely assessed (either in part or in its entirety) in the remaining individuals (e.g., due to stricturing disease of the colon or ileocecal valve). Using endoscopy as the reference standard, MRE was truly positive in 34 patients, falsely positive in 8 patients, truly negative in 18 patients, and falsely negative in 17 patients. Thus, the sensitivity and specificity of MRE for detecting endoscopic ulcers were 66.7% (95% CI, 52.1–79.2%) and 69.2% (95% CI, 48.2–85.7%), respectively. The positive predictive value and negative predictive value were 81.0% (95% CI, 69.8–88.6%) and 51.4% (39.9–62.8%), respectively.

## Discussion

Our study demonstrates that radiologic ulcers are frequently visible on clinical MRE examinations in children with newly diagnosed ileal CD. While commonly present, there was considerable disagreement between experienced radiologists for establishing their presence. Our results also suggest that the presence of these ulcers is associated with a more active disease, including higher CRP and fecal calprotectin levels, greater bowel wall thickening, and longer length of disease, on average. To date, there is a paucity of literature correlating the presence of radiologic ulcers with other MRI features of intestinal active inflammation, clinical markers of intestinal active inflammation such as laboratory testing or patient-reported outcomes, or meaningful clinical outcomes, such as a treatment response and need for future surgery, particularly in children.

Radiologic ulcers are increasingly recognized as a key finding of active intestinal inflammation on MRE examinations. Their increased detection may be due to increased radiologist awareness as well as improving image quality over time, including higher spatial resolution imaging and decreased motion artifacts due to faster imaging techniques. In an early study, Rimola et al. showed an association between radiologic ulcers and bowel wall active inflammation, and thus included this imaging finding in the original Magnetic Resonance Index of Activity (MaRIA) [5]. In their scoring system, the presence of radiologic ulcers was weighted twice that of bowel wall edema. More recently, Ordás et al. also included radiologic ulcers in the simplified MaRIA [6]. In their scoring system, the presence of ulcers was again weighted twice that of other included MRE features, including bowel wall thickening, bowel wall edema, and perienteric inflammation. Radiologic ulcers were also included in the MONITOR index which has been shown to predict recurrent CD in the presence of an ileocolonic anastomosis [8]. In their study, all included MRE findings had a weighting of 1 except for radiologic ulcers, which received a weighting of 2.5 reflecting a higher adjusted odds ratio. Buisson et al. also included the presence of ulcers in a validated scoring system for assessing treatment response and transmural healing [9]. More recently, the multinational ImageKids study developed the Pediatric Inflammatory Crohn’s Magnetic Resonance Enterography Index (PICMI), which also included radiologic ulcers along with bowel wall thickening, bowel wall restricted diffusion, mesenteric edema, and the comb sign in its final validated form [10].

There is limited data showing agreement between radiologists for identifying radiologic ulcers, particularly in children. Like our study, Rees et al. showed fair agreement (*k* = 0.37) between five fellowship-trained pediatric radiologists for detecting the presence of radiologic ulcers in children with CD [11]. In a study of adult patients with CD, Tsai et al. found no agreement between two fellowship-trained abdominal radiologists for detecting the presence of radiologic ulcers, with a *k* statistic less than 0 indicating agreement worse than that expected due to chance [12]. However, in another adult study by Jairath and colleagues, ulceration of the terminal ileum demonstrated better inter-rater agreement (ICC = 0.60), whereas agreement for the colon and rectum was worse with ICCs ranging from 0.09 to 0.55 depending on location [4].

Interestingly, radiologists in our study demonstrated a higher level of agreement for ulcer extent (i.e., number of radiologic ulcers) compared to either presence of ulcers or ulcer severity (i.e., ulcer depth). The overall low levels of agreement related to radiologic ulcer assessment in our study, particularly when compared to other MRI findings of active disease, suggest that this imaging finding may not have sufficient reliability to be used in clinical practice at this time. It is conceivable that standardized and validated definitions could improve agreement between radiologists. Furthermore, additional radiologist training related to this imaging finding could improve detection and inter-radiologist agreement. Finally, maximizing bowel distention is likely to enhance the radiologist’s sensitivity and specificity in detecting ulcers. However, further research is needed to confirm this.

Our study has limitations. First, this was a retrospective investigation performed at a single institution. Second, as this study only included children, our results, including the frequency of radiologic ulcers at the time of diagnosis, may not be generalizable to adults. Third, there remains a lack of accepted standardized definitions for what constitutes a radiologic ulcer and how to define ulcer severity and extent. Finally, while our study did evaluate the diagnostic performance of MRE compared to endoscopy, endoscopy reports remain unstandardized in the clinical setting with regard to terminology (e.g., erosion vs. aphthous lesion vs. superficial ulcer).

Our study confirms that radiologic ulcers are common in children with newly diagnosed ileal CD and are visible at MRE, although inter-radiologist agreement is only fair and diagnostic performance is less than ideal. The presence of ulcers seems to indicate more severe active inflammation and is associated with both clinical laboratory markers and other MRE findings of disease activity. The relatively low degree of inter-radiologist agreement suggests the need for more precise definitions for determining the presence, severity, and extent of radiologic ulcers as well as a need for more formal radiologist training. Further research also is needed to understand if the presence of radiologic ulcers (both alone and in combination with other imaging features) correlates with important clinical outcomes in children, such as treatment response and future need for surgery.

## Supplementary Information

Below is the link to the electronic supplementary material.

Supplementary file1 (DOCX 17 KB)

## Data Availability

Study data are available upon reasonable request.
