# Supplementary material for: MR enterography radiologic ulcers in newly diagnosed ileal Crohn disease in children: frequency, inter-radiologist agreement, and clinical correlation
Source: Pediatr Radiol. 2024 Sep 18;54(11):1842–9. doi: 10.1007/s00247-024-06056-7 (PMC11473662; doi:10.1007/s00247-024-06056-7)
Supplement: Supplementary file 1 — Supplementary file1 (DOCX 17 KB) [file 247_2024_6056_MOESM1_ESM.docx]

**Supplementary information**

**Supplemental Table** Institutional clinical MR enterography protocol at 1.5-T

| **Sequence** | **Excitation**  **Flip Angle** | **Slice Thickness / Gap (mm)** | **Echo Time (ms)** | **Repetition Time (ms)** | **Number of Signal Averages** | **b-values (s/mm^2^) / Averages** | **Fat Suppression Method** |
| --- | --- | --- | --- | --- | --- | --- | --- |
| Coronal SSFSE | 90 | 5 / 1 | 80 | NA | 1 | NA | NA |
| Axial SSFSE | 90 | 5 / 1 | 80 | NA | 1 | NA | NA |
| Axial SSFSE with fat-saturation | 90 | 5 / 1 | 80 | NA | 1 | NA | SPAIR |
| Axial diffusion-weighted imaging | 90 | 6 / 1 | Minimum (~62) | 3000 | 1 | 0 /2, 100 /2, 800 / 4 | SPIR |
| Coronal precontrast 3D T1-weighted* | 15 | 5^#^ | Minimum (both echoes) | Minimum | 1 | NA | Dixon |
| Coronal postcontrast 3D T1-weighted | 15 | 5^#^ | Minimum (both echoes) | Minimum | 1 | NA | Dixon |
| Axial postcontrast 3D T1-weighted | 15 | 5^#^ | Minimum (both echoes) | Minimum | 1 | NA | Dixon |

NA=not applicable; SPAIR=spectral adiabatic inversion recovery; SPIR=spectral presaturation with inversion recovery; SSFSE=single-shot fast spin-echo

*3D two-echo radiofrequency spoiled gradient echo Dixon sequence

^#^reconstructed at 2.5 mm increments
